# Phytochemical-mediated efferocytosis and autophagy in inflammation control

**DOI:** 10.1038/s41420-024-02254-2

**Published:** 2024-12-18

**Authors:** Asma Vafadar, Amir Tajbakhsh, Fatemeh Hosseinpour-Soleimani, Amir Savardshtaki, Mohammad Hashem Hashempur

**Affiliations:** 1 Department of Medical Biotechnology, School of Advanced Medical Sciences and Technologies, Shiraz University of Medical Sciences, Shiraz, Iran; 2 Student Research Committee, Shiraz University of Medical Sciences, Shiraz, Iran; 3 Pharmaceutical Sciences Research Center, Shiraz University of Medical Sciences, Shiraz, Iran; 4 Department of Applied Cell Sciences and Tissue Engineering, School of Advanced Medical Sciences and Technologies, Shiraz University of Medical Sciences, Shiraz, Iran; 5 Infertility Research Center, Shiraz University of Medical Sciences, Shiraz, Iran; 6 Research Center for Traditional Medicine and History of Medicine, Department of Persian Medicine, School of Medicine, Shiraz University of Medical Sciences, Shiraz, Iran

**Keywords:** Cell death and immune response, Cell death, Natural products

## Abstract

Efferocytosis, the clearance of apoptotic cells, is a critical process that maintains tissue homeostasis and immune regulation. Defective efferocytosis is linked to the development of chronic inflammatory conditions, including atherosclerosis, neurological disorders, and autoimmune diseases. Moreover, the interplay between autophagy and efferocytosis is crucial for inflammation control, as autophagy enhances the ability of phagocytic cells. Efficient efferocytosis, in turn, regulates autophagic pathways, fostering a balanced cellular environment. Dysregulation of this balance can contribute to the pathogenesis of various disorders. Phytochemicals, bioactive compounds found in plants, have emerged as promising therapeutic agents owing to their diverse pharmacological properties, including antioxidant, anti-inflammatory, and immunomodulatory effects. This review aims to highlight the pivotal role of phytochemicals in enhancing efferocytosis and autophagy and explore their potential in the prevention and treatment of related disorders. This study examines how phytochemicals influence key aspects of efferocytosis, including phagocytic cell activation, macrophage polarization, and autophagy induction. The therapeutic potential of phytochemicals in atherosclerosis and neurological diseases is highlighted, emphasizing their ability to enhance efferocytosis and autophagy and reduce inflammation. This review also discusses innovative approaches, such as nanoformulations and combination therapies to improve the targeting and bioavailability of phytochemicals. Ultimately, this study inspires further research and clinical applications in phytochemical-mediated efferocytosis enhancement for managing chronic inflammatory and autoimmune conditions.

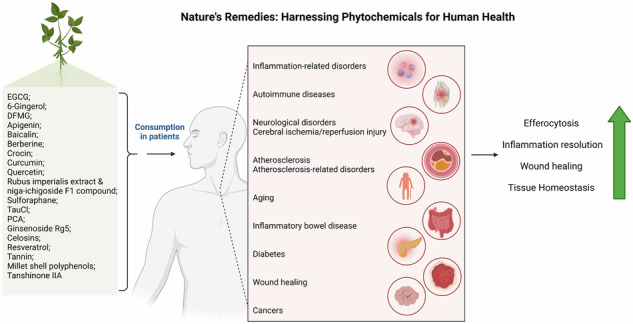

## Facts

- Dysregulation of balance between autophagy and efferocytosis can contribute to the pathogenesis of various disorders.
- Phytochemicals derived from natural sources can enhance efferocytosis and potentially treat the related conditions.
- Different phytochemicals, such as flavones, flavonoids, and anthocyanidins have anti-inflammatory effects and can prevent various disorders.
- EGCG, Baicalin, 6-Gingerol, Apigenin, and Berberine promote efferocytosis and inhibit inflammation.
- The synergistic effects of combining multiple phytochemicals could lead to enhanced anti-inflammatory and efferocytosis-promoting effects, warranting systematic studies on optimal combinations.

## Open Questions

1. What are the specific molecular mechanisms by which different phytochemicals enhance efferocytosis and autophagy in various cell types, and how do these mechanisms differ between phytochemicals?
2. How can the findings regarding phytochemical effects on efferocytosis and autophagy be translated into clinical practice for treating autoimmune and inflammatory disorders, and what specific clinical trials are needed to evaluate their efficacy?
3. What are the potential synergistic effects of combining different phytochemicals on efferocytosis and autophagy, and how might these combinations be optimized for therapeutic use?
4. What are the long-term effects of phytochemical supplementation on efferocytosis and autophagy in chronic inflammatory conditions, and how do these effects impact overall disease progression and patient outcomes?

## Introduction

**Fig. 1:**
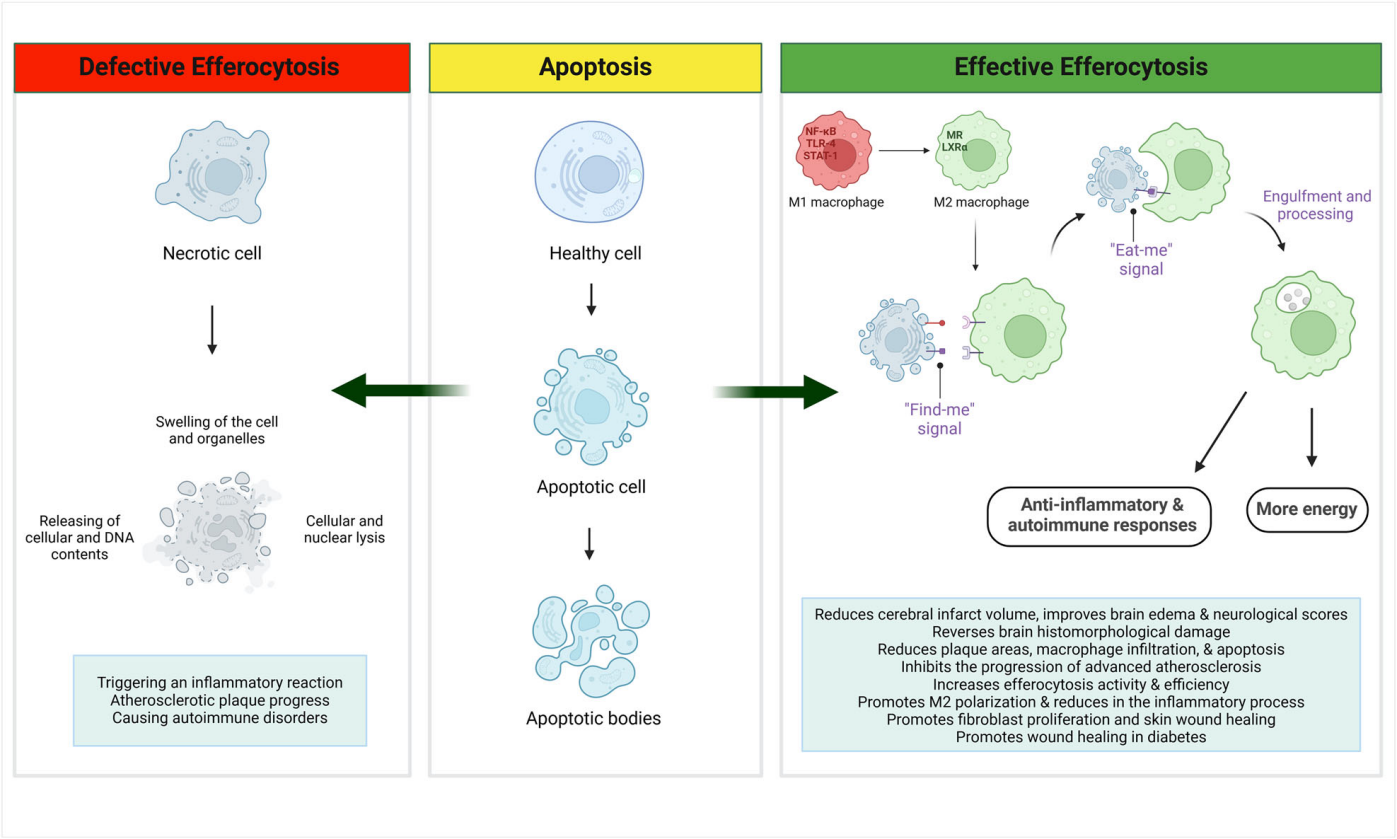
Efferocytosis mechanisms and their implications. An illustration depicting effective and defective efferocytosis with their consequences.

Every day, approximately 0.4% of an adult human’s estimated 37.2 trillion cells die [1]. Maintaining homeostasis in multicellular organisms is a crucial process that relies on cell death, and efficient removal of dying cells. In almost all physiological and pathological situations, a controlled series of signaling events (regulated cell death) triggers cells to participate in their demise [2]. The clearance of dead cells is crucial for maintaining normal bodily functions as well as preventing diseases. Efferocytosis, the process of removing apoptotic cells (ACs) and non-inflammatory phagocytic activity by both professional and non-professional phagocytes, is required for tissue homeostasis during physiological function and restoration of homeostasis after disorders [3, 4] (Fig. 1). Efferocytosis becomes defective in several non-resolving, chronic inflammatory diseases, leading to the accumulation of ACs [5, 6]. In this case, the release of cellular and inflammatory mediators can trigger autoimmune and inflammatory disorders, such as atherosclerosis, autoimmune diseases, and neurodegenerative conditions. The failure to efficiently clear dead cells disrupts tissue homeostasis and promotes pathological inflammation [7, 8]. Abnormal efferocytosis is linked to different autoimmune and inflammatory disorders, such as acute lung injury (ALI, a clinical syndrome characterized by widespread inflammation and increased permeability of the alveolar-capillary barrier) rheumatoid arthritis (RA), asthma, autoimmunity lymphoproliferative syndrome (ALPS), infection, diabetes, systemic lupus erythematosus (SLE), multiple sclerosis (MS), and other inflammatory conditions [8–13]. During inflammation, the ability of phagocytes to engulf ACs is inhibited by the generation of reactive oxygen species (ROS) by neutrophils. ROS triggers the activation of RhoA, a GTPase that acts as a negative regulator of efferocytosis, leading to a decrease in the engulfment of ACs by neighboring cells [14–16]. Rho GTPases regulate efferocytosis by controlling the actin cytoskeleton, which is essential for phagocyte motility and AC engulfment. In addition, Rac1, a member of the Rho GTPase family, plays a central role in regulating the actin cytoskeleton and signaling pathways involved in efferocytosis [17, 18] (Table 1).

**Table 1:** The main steps in the efferocytosis process.

Num	Steps	Factors and pathways
1	Recruitment of phagocytes [42, 174–177]	-Phagocytes are guided towards ACs by “Find-Me” signals, which act as chemoattractants. -“Find-Me” signals, such as fractalkine, LPC, S1P, and nucleotides (ATP and UTP), play a crucial role in guiding phagocytes toward ACs. -These signals not only guide phagocytes but also enhance their ability to clear ACs and trigger anti-inflammatory responses (detailed in step 5).
2	Recognition of ACs [44, 178–182]	-Phagocytes distinguish between phagocytic and non-phagocytic cells through the interaction of ACs’ ligands (“Eat-Me” signals) with receptors present in phagocytes. -Different ligands present on the surface of ACs, such as PS, ox-LDL, calreticulin (Calr), ICAM3, C1q, and annexin I, are recognized by engulfment receptors on phagocytes. -Phagocytes can also recognize healthy cells through the “Don’t Eat-Me” signal, which includes CD31, CD47, CD24, PD-L1, and MHC I, to prevent the engulfment of healthy cells.
3	Internalization of ACs [183–193]	-Rac1, a Rho family GTPase, plays a key role in the internalization of ACs during efferocytosis. -Engulfment receptors initiate downstream signaling pathways that trigger Rac1, leading to cytoskeletal rearrangement and engulfment of the target. -Different PS receptors, such as BAI1 and Stabilin-2, have established signaling pathways downstream. -Tim-4 acts as a tethering receptor and promotes efferocytosis by binding ACs on phagocytes, which then use other engulfment receptors, such as integrin, to ingest the ACs. This process is known as two-step engulfment. -Tethering receptors like Tim-4 can promote efferocytosis without interacting with their co-receptors, but they may have biochemical interactions with co-receptors, such as Mertk.
4	Degradation of ACs [44, 97, 194–202]	Phagocytosis degrades ACs by creating phagosomes that become more acidic and eventually join with lysosomes for AC collapse. -LAP accelerates the process by maturing phagosomes more efficiently, leading to faster AC degradation. -Phagocytes experience a doubling of intracellular contents during efferocytosis, leading to the release of some contents and adjustments in metabolism to maintain appropriate levels. -Engulfment receptors recognizing PS and AC-derived sterols activate LXR and PPAR, resulting in altered energy metabolism and cholesterol efflux. -Alterations in energy metabolism impact the body’s ability to fight inflammation by promoting the generation of anti-inflammatory cytokines and altering glucose transport and lactate release during efferocytosis.
5	Anti-inflammatory responses [203, 204]	-Efferocytosis is associated with an anti-inflammatory response that involves the production of anti-inflammatory cytokines (IL-10 & TGF-_β_) and the suppression of pro-inflammatory cytokines (TNF-α & IL-1_β_). -Nuclear receptors (*e.g*., LXR & PPAR) are activated by binding to AC-derived ligands, leading to the upregulation of genes involved in efferocytosis and anti-inflammatory responses (*e.g*., lipid metabolism & anti-inflammatory responses).

*ACs* Apoptotic cells, *BAI1* Brain-specific angiogenesis inhibitor 1, *CD* Cluster of differentiation, *ICAM3* Intercellular adhesion molecule 3, *IL* Interleukin, *LAP* LAP-associated phagocytosis, *LDL* Low-density lipoprotein, *LPC* Lysophosphatidylcholine, *LXR* Liver X receptor, *MHC* Major histocompatibility complex, *PD-L1* Programmed death ligand 1, *PPAR* Peroxisome proliferator-activated receptors, *PS* Phosphatidylserine, *S1P* Sphingosine 1-phosphate, *Tim-4* T-cell membrane protein 4, *TGF-beta* Transforming Growth Factor-β, *TNF- α* Tumor necrosis factor-alpha.

Antioxidants derived from natural sources have significant bioactivities that can be used to prevent or cure diseases related to oxidative stress. These antioxidants work by reducing or scavenging ROS, inhibiting lipid peroxidation, and chelating free metal ions. Moreover, phytochemicals with antioxidant properties increase efferocytosis and have the potential to treat efferocytosis-related conditions [19–21]. Numerous studies published over the recent have indicated the antioxidant and anti-inflammatory effects of various phytochemical nutrients, such as flavones, flavonoids, flavanols, isoflavones, and anthocyanidins. Phytochemicals possess bioactive properties that can prevent or treat an extremely wide array of diseases [22–28].

Phytochemicals are natural compounds found in plants that have been studied for their health benefits and possible therapeutic uses. There are several advantages to using phytochemicals compared with other treatments. These compounds come from natural sources, such as fruits, vegetables, herbs, and spices, which many people prefer because they fit well with a holistic view of health and wellness. Phytochemicals offer a wide range of health benefits due to their different chemical structures. They can act as antioxidants, reduce inflammation, fight microbes, and even help prevent cancer. Additionally, phytochemical treatments have fewer side effects than conventional medications. Foods rich in phytochemicals also provide important nutrients, vitamins, minerals, and dietary fiber, all of which contribute to overall health. However, it’s important to note that the effectiveness of phytochemical treatments are can depend on factors, such as the specific compound, the amount taken, how it’s prepared, individual differences, and the health issue being addressed [29, 30]. The aim of this review was to comprehensively examine the role of phytochemical antioxidants in modulating efferocytosis and their potential therapeutic applications for autoimmune and inflammatory disorders. Furthermore, this review explored the mechanisms by which phytochemicals modulate efferocytosis, including their effects on ROS generation, RhoA activation, and phagocytic clearance. The association between abnormal efferocytosis and various autoimmune and inflammatory conditions. Ultimately, the review consolidated the existing knowledge, identified research gaps, and offered insights into the therapeutic potential of phytochemicals against efferocytosis-related disorders.

## Efferocytosis: a key process in immune regulation and tissue homeostasis

The immune response differs significantly between the two primary types of cell death, apoptosis (planned cell death) and necrosis (random cell death). Phagocytosis, the process by which cells engulf and digest cellular debris, is initiated by efferocytosis (the clearance of ACs) without inducing an inflammatory or immune response. Efferocytosis is believed to be anti-inflammatory and promote immune tolerance, meaning that the immune system does not exhibit a strong reaction. Necrosis, on the other hand, happens when cells die prematurely as a result of external causes, resulting in a pro-inflammatory and immunostimulatory response [31–33]. The failure to clear ACs can lead to necrotic features in ACs, causing autoimmune disorders over time through the stimulation of pro-inflammatory pathways [11, 34, 35].

Apoptosis occurs daily in several cells, and is part of normal development and tissue maintenance. To prevent the release of cytotoxic materials into the environment, it is important to remove these ACs quickly. Efferocytosis is the process by which cellular corpses are removed [36] (Fig. 1). Since the 1990s, this process has been of interest and involves complicated molecular relationships and a variety of phagocytosis and efferocytosis pathways [37–39]. AC clearance is an important process that helps maintain and heal homeostasis after injury. When ACs are not efficiently cleaned, their membrane integrity is compromised, leading to intracellular content leakage and secondary necrosis. Efferocytosis is the rapid removal of ACs from tissues by efferocytes to prevent subsequent necrosis [40].

Efferocytosis involves five steps: attraction of phagocytes to ACs, identification of ACs by phagocytes, internalization of ACs into phagocytes, degradation of ACs, and anti-inflammatory responses [41] (Table 1). The process of efferocytosis requires coordination between three signals: “Find-Me”, “Eat-Me”, and “Don’t Eat-Me”, in order to maintain equilibrium. Various cell types can participate in efferocytosis, including professional phagocytes, such as immature dendritic cells and macrophages, as well as nonprofessional cells, such as epithelial cells, fibroblasts, and endothelial cells [42–44] (Fig. 1). Macrophages, as professional phagocytes, play an important role in enabling successful efferocytosis. Generally, the signals recognized as valuable surface markers are found on ACs that facilitate the proper performance of phagocytic processes [39].

Efferocytosis plays a crucial role in maintaining homeostasis in biological systems (Table 1). It is essential for human health because it helps to avoid the negative consequences of cell death, guarantees the integrity of tissues and organs and fosters a healthy immunological response. Efferocytosis is a critical process that maintains tissue homeostasis by clearing ACs, suppressing inflammation, promoting self-tolerance, and activating resolution pathways. The regulation of macrophage polarization plays a key role in the successful execution of efferocytosis.

The failure to effectively clear dead cells results in the buildup of uncleare dead cells. These cells then release pro-inflammatory molecules, which in turn leads to an excessive inflammatory response. This highlights the crucial role of clearing ACs in maintaining homeostasis. Consequently, any changes in the ability of phagocytes, whether professional or non-professional, to engulf ACs can have an impact on tissue homeostasis. This condition can potentially trigger inflammation and/or developmental abnormalities, depending on the specific tissue [3, 13, 45]. Efferocytosis has three critical effects in addition to avoiding additional cell death: it inhibits inflammatory responses, promotes self-tolerance, and activates pathways that help resolve the problem. Efferocytosis promotes the generation of anti-inflammatory and tissue-repair chemicals, whereas deficient efferocytosis can result in excessive inflammation and various diseases [46]. The clearance of ACs necessitates the presence of receptors on the phagocytes that can identify the ligands associated with ACs. Additionally, phagocytes must undergo cytoskeletal reorganization to engulf ACs bound to other cells [41]. The elimination of ACs by macrophages is achieved through the generation of specific molecules produced during the degradation of ACs within the phagolysosome. Furthermore, the fusion of phagosomes with lysosomes is required to degrade ACs contents. When macrophages ingest ACs, they regulate the production of pro-inflammatory cytokines, suppressing their release while increasing the production of molecules that mitigate inflammation and facilitate resolution and repair [47, 48]. These processes are carried out through pathways that are both integrated and mechanistically distinct. AC interaction with the efferocyte receptor T cell immunoglobulin mucin receptor 1 (TIM1), for example, reduces the generation of TNF, CC-chemokine ligand 5 (CCL5), and interleukin (IL)-6 by blocking the activation of NF-κB. AC binding to the efferocyte receptor stabilin 2, on the other hand, causes the synthesis of transforming growth factor beta (TGF_β_) [49, 50]. ACs block Toll-like receptor (TLR) as well as type 1 interferon (IFN)-mediated pro-inflammatory signaling pathways by interacting with the MertK and AXL [51]. ACs inhibit the function of IκB kinase (IKK), thereby hindering the activation of TLR4-induced, NF-κB-dependent TNF expression. On the other hand, AXL activation leads to an increase in the levels of Twist-related proteins, which act as transcriptional repressors that suppress TLR4-induced TNF promoter activity. Additionally, these receptors on efferocytes upregulate the expression of the E3 ubiquitin ligase suppressors of cytokine signaling 1 (SOCS1) and SOCS3. The upregulation of SOCS1 and SOCS3 inhibits the signaling of signal transducer and activator of transcription 1 (STAT1) mediated by IFNα, consequently blocking pro-inflammatory gene expression [52–54]. Sterols are transported to efferocytes after the degradation of phagolysosomal AC. Once delivered, these sterols activate nuclear sterol receptors (e.g., liver X receptor-alpha (LXRα), and peroxisome proliferator-activated receptor (PPAR)γ, PPARδ). When these receptors are activated, anti-inflammatory cytokines, such as IL-10 and TGF_β_ are produced. Furthermore, it induces the differentiation of T_regulatory_ cells and T_helper_ 2 cells, thereby aiding in the resolution of immune responses [55–57]. Moreover, the nuclear receptor corepressor is bound by PPARγ and LXRs, thereby preventing its removal from the promoter sequences of genes encoding specific pro-inflammatory cytokines (e.g. IL-1β and TNF) [58, 59]. Furthermore, the interaction between efferocytes and ACs promotes the generation of specific pro-resolving mediators while reducing the formation of pro-inflammatory leukotrienes. The activation of the AC receptor MertK causes the translocation of lipoxygenase 5 from the nucleus to the cytoplasm, thereby increasing the production of lipoxin A4. These combined activities effectively suppress inflammation and enhance the resolution of inflammation [48, 60].

Any deficiency in this process is linked to several conditions, including inflammatory, autoimmune, and atherosclerosis conditions [5, 11, 61]. When phagocytes, such as macrophages, fail to destroy ACs quickly owing to cell membrane breakdown, they change into secondary necrotic cells (NCs). As a result, the release of cellular and DNA from these NCs into the environment might trigger an inflammatory reaction. The precise control of macrophage polarization is crucial for efficient efferocytosis in both pathological and physiological contexts. Therefore, understanding and regulating this polarization are of significant importance [45]. The activation state and functional characteristics of macrophages, depending on the condition, determine the emergence of two distinct phenotypes: M1 (classically activated) and M2 (alternatively activated) [62, 63]. The polarization of macrophages is influenced by inflammatory parameters. Pro-inflammatory cytokines, such as IL-12, pathogen-associated molecular patterns (PAMPs), TNF-α, and IFN-ϒ, such as lipopolysaccharide (LPS), polarize M1 macrophages. In macrophages, these stimuli efficiently promote the M1 phenotype. The notable actions of M1 macrophages (pro-inflammatory) include the secretion of TNF-α, IL-6, and IL-1, which contribute to inflammatory responses and elevation of type-1 Th1 cell responses. Furthermore, M1 macrophages have tumoricidal properties [63] (Table 3).

Overall, macrophage polarization favors the M1 phenotype for the attack of intracellular infections and enhancement of anticancer effects. Furthermore, M2 macrophages are known anti-inflammatory cells that express high levels of IL-10 and low levels of pro-inflammatory cytokines. These qualities are important in a variety of physiological processes, including wound healing and inflammation control. Inflammatory M1 macrophages are present in the early stages of wound healing. In this respect, M1 macrophages and neutrophils work together to eliminate pathogens and debris. Subsequently, apoptotic neutrophils are absorbed by inflammatory M1 macrophages during the efferocytosis process [63]. M1 macrophages produce nitric oxide (NO), ROS via inducible nitric oxide synthase (iNOS), and pro-inflammatory cytokines, such as IL-1, IL-12, IL-23, and TNF when activated. M2 macrophages (anti-inflammatory), on the other hand, release anti-inflammatory cytokines and growth factors, such as IL-10, IL-4, and TGF_β_, as well as angiogenic and pro-fibrotic characteristics [13, 64].

## Autophagy and efferocytosis

**Fig. 2:**
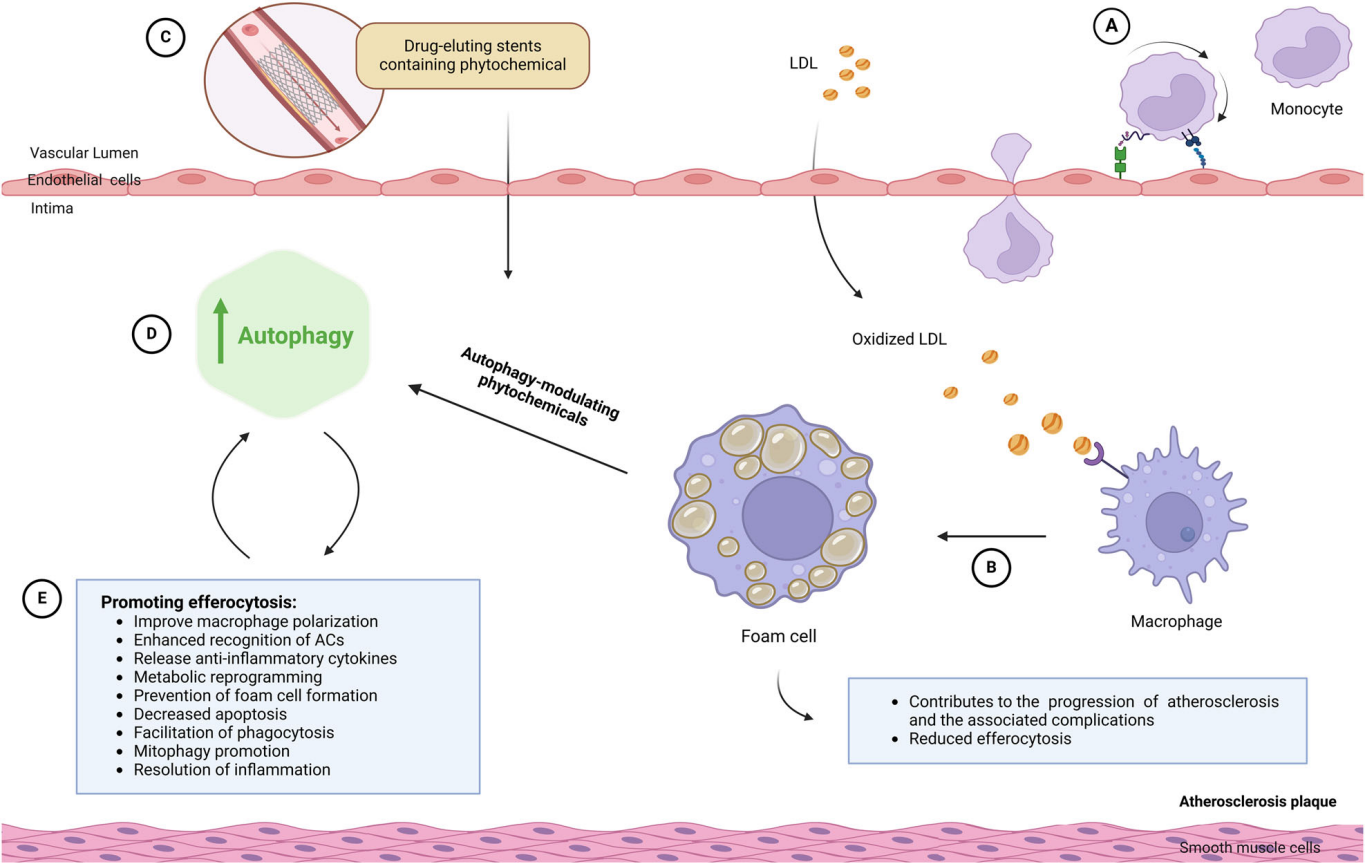
The modulation of effective autophagy and efferocytosis in foam cell/ lipid-laden macrophage by phytochemicals in atherosclerosis plaque. **A** The entry of monocytes into the intima. Monocytes migrate from the bloodstream into the innermost layer of the artery wall, called the intima. Once in the intima, the monocytes differentiate into macrophages. **B** illustrates the formation of foam cells. In intima, macrophages start to accumulate modified lipoproteins, such as oxidized low-density lipoprotein (Ox-LDL), leading to the formation of foam cells. The accumulation of these foam cells, along with other cellular debris and extracellular matrix, leads to the formation of an atherosclerotic plaque within the intima of the artery wall. In this case, this cell can modulate by normal autophagy *via* phytochemicals. **C**, **D** shows the effect of drug-eluting stents containing phytochemicals, leading to an increase in autophagy. **E** This illustrates that improved autophagy induced by phytochemicals can promote efferocytosis, and conversely, increased efferocytosis can enhance autophagy. Ultimately, this balance fosters an anti-inflammatory environment, reduces atherosclerotic plaque development, and improves plaque stability. Thus, by incorporating phytochemicals, the drug-eluting stents could potentially provide added therapeutic benefits beyond the currently used pharmaceutical drugs, leading to improved long-term outcomes for patients with atherosclerotic cardiovascular disease. Additional details about the intricate factors and pathways connecting autophagy and efferocytosis, along with specific phytochemicals that can regulate this balance, can be found in Table 2.

The interplay between autophagy and efferocytosis is crucial for maintaining cellular homeostasis and preventing inflammation. Autophagy is a highly conserved cellular process that serves as a crucial pathway for the clearance of damaged organelles and misfolded proteins. Importantly, autophagy has been shown to play a key role in the efficient clearance of ACs by activated inflammatory cells, such as neutrophils and macrophages [65, 66]. Autophagy is integral to macrophage function and efferocytosis. By regulating polarization, cytokine secretion, metabolism, and phagocytosis, autophagy not only supports macrophage survival and function but also promotes the resolution of inflammation and maintenance of tissue homeostasis through the effective clearance of ACs [67] (Fig. 2).

Autophagy significantly influences the polarization of macrophages into various functional states, including M1 and M2 types [68, 69]. M2 macrophages exhibit greater efficiency in efferocytosis, facilitating tissue repair and the resolution of inflammation. Through the regulation of polarization, autophagy enhances the capacity of macrophages to recognize and clear ACs. Additionally, it affects the secretion of various cytokines that regulate immune responses. By promoting the release of anti-inflammatory cytokines, autophagy fosters a favorable environment for efferocytosis, thereby enhancing the ability of macrophages to engulf and digest ACs [69, 70].

IL-10 inhibits the mechanistic target of rapamycin (mTOR) activation through the signal transducer and activator of transcription 3- DNA damage-inducible transcript 4 (STAT3-DDIT4) pathway, which promotes mitophagy and oxidative phosphorylation while suppressing aerobic glycolysis. In contrast, the mTOR signaling pathway facilitates the shift toward glycolysis necessary for M1 macrophages by increasing HIF-1 expression [70]. mTOR is a key regulator of autophagy, that suppresses the autophagy process. When mTOR is active, it inhibits autophagy, thereby preventing the degradation of cellular components. This regulation helps maintain cellular homeostasis under nutrient-rich conditions, but it can hinder autophagy during periods of stress or nutrient deprivation [71, 72]. Adenosine Monophosphate-activated protein kinase (AMPK) also plays a role in regulating M2 polarization of macrophages. By activating autophagy and promoting metabolic reprogramming, AMPK supports the transition of macrophages toward the M2 phenotype, which is associated with anti-inflammatory responses and tissue repair. This regulation enhances the ability of macrophages to clear ACs and secrete anti-inflammatory cytokines, contributing to the resolution of inflammation and overall tissue homeostasis [73]. Thus, autophagy plays a vital role in cellular metabolism by providing energy and recycling cellular components [70]. This metabolic flexibility allows macrophages to adapt to different functional states, thereby supporting their efficiency in performing efferocytosis, particularly under stress conditions. Furthermore, autophagy enhances the machinery responsible for recognizing and engulfing ACs, facilitating the phagocytosis process and ensuring effective clearance of dead cells while preventing secondary necrosis [67].

Numerous studies have highlighted the close association between impaired autophagy and decreased efferocytosis, or the phagocytic clearance of ACs, in the context of various inflammatory disorders, such as atherosclerosis, inflammatory bowel disease (IBD), and neurodegenerative diseases [65, 66, 74–79] (Table 2).

**Table 2:** The effect of phytochemicals on efferocytosis and autophagy.

Phytochemical	Sources	Effects on Efferocytosis & inflammation resolution	Related to efferocytosis	Conditions	Tested models
EGCG [109, 205]	*Camellia sinensis* (Green tea)	-Attenuates inflammatory response by targeting Notch signaling pathway -Knockdown of Notch 1/2 expression impairs the downregulation of inflammatory response by EGCG -Inhibits LPS-induced inflammation & turns off Notch signaling -Enhances phagocytosis of macrophages & populations of T- & B-cells & NK cell activity.	-The inhibition of the Notch 1 & Notch 2 attenuates inflammation. -Notch signal regulates macrophage phagocytosis of tumor cells by SIRPα.	Inflammation, Leukemia	-Human macrophages, -Leukemic BALB/c mice
6-Gingerol [74, 75]	*Zingiber officinale* (Ginger)	-Reduces cerebral infarct volume, improves brain edema & neurological scores, & reverses brain histomorphological damage. -Reduces NLRP3 inflammasome-derived inflammation & neuronal apoptosis, & upregulates autophagy. -Has protective effects against 6-hydroxydopamine-induced apoptosis.	Autophagy & efferocytosis are interconnected processes, where autophagy boosts the ability to perform efferocytosis, & efferocytosis influences autophagic pathways.	Cerebral ischemia/reperfusion injury	-Adult male Sprague-Dawley rats
Apigenin [206]	*Petroselinum crispum* (Parsley), *Matricaria chamomilla* (Chamomile), *Apium graveolens* (Celery), *Citrus × sinensis* (Orange)	-Induces apoptosis of Ox-LDL-loaded macrophages -Reduces PAI-2 expression -Suppresses phosphorylation of AKT at Ser473 -Executes anti-atherogenic effects by inducing macrophage apoptosis	-Uptake of Ox-LDL by macrophages can impair their efferocytic function, as Ox-LDL can contribute to cellular stress & dysfunction. -Increased expression of PAI-2 in Ox-LDL-loaded macrophages may be a protective response to maintain their efferocytic capabilities. -PAI-2 inhibits proteases involved in the breakdown of extracellular matrix, which is important for the efficient engulfment of ACs during efferocytosis. -AKT signaling enhances macrophage survival & efferocytosis by supporting cytoskeletal rearrangements & phagocytosis of ACs.	Atherogenesis	*ApoE*^*-/-*^ mice (in vivo) Ox-LDL-loaded MPMs (in vitro)
Baicalin [23, 207–209]	*Scutellaria baicalensis* (Baikal skullcap)	-Inhibits the activation of the CX3CL1-CX3CR1 axis & NF-κB pathway. -Induces M2C macrophage polarization, enhancing phagocytosis & efferocytosis. -Impairs Th1 polarization by inhibiting DC maturation & suppressing the expression of pro-inflammatory molecules. -Alleviates IBD by limiting M1 macrophage polarization, & promoting anti-inflammatory cytokine expression.	-The CX3CL1-CX3CR1 axis is involved in inflammation, & its inhibition, along with the NF-κB pathway, can suppress pro-inflammatory signaling. -Polarization towards the M2C phenotype enhances the phagocytic & efferocytic functions of macrophages. -Impairs Th1 polarization by inhibiting DC maturation & suppressing pro-inflammatory molecule expression. -Th1 cells promote pro-inflammatory responses.	-ALI; -IBD; -DCs-related acute & chronic diseases.	-LPS-induced ALI in mice CX3CL1-knockout (CX3CL1-KO or *CX3CL1*^*-/-*^) mice; - MPMs, -Mice with DSS-induced colitis;
Berberine [122, 123]	*Hydrastis canadensis* (Goldenseal), *Berberis vulgaris* (Barberry), *Coptis chinensis* (Chinese goldthread)	-Reduces ox-LDL-induced inflammation in a dose- & time-dependent manner. -Increases the ratio of LC3II/LC3I & SQSTM1/p62, which are markers of autophagy activation. -Increases the ratio of the activated form of AMPK & decreases the ratio of activated form of mTOR.	- Ox-LDL can trigger inflammation, impairing efferocytosis. -An increased LC3II/LC3I & SQSTM1/p62 ratio indicates enhanced autophagy activation, engulfment & degradation of ACs & promoting efferocytosis. -The increased ratio of activated AMPK & decreased ratio of activated mTOR indicate a shift towards a pro-autophagy state, enhancing the autophagic machinery & efferocytosis.	- Atherosclerosis, inflammation induced by ox-LDL; -Bacterial infection	-J774A.1 cells; -Murine macrophages
Crocin [210]	*Crocus sativus* (Saffron)	-Elevates M1 activity indicators in uncommitted macrophages -Prevents the increase in M1 indicators when co-treated with LPS + IFN-γ -Increases M1 induction when pretreated before the addition of LPS + IFN-γ -IL-10 was not detectable in any experimental groups	- Preventing the increase in M1 indicators with LPS + IFN-γ co-treatment, may support a more anti-inflammatory environment conducive to efferocytosis. -The absence of IL-10 suggests weak anti-inflammatory signaling, which may impede inflammation resolution & the clearance of ACs.	M1/M2 macrophage imbalance, inflammation	J774A.1 macrophages
Hydroalcoholic Fruit Extract of Solanum diploconos (Mart.) Bohs [211]	Fruit of the *Solanum diploconos* (Holy blackberry)	-Impairs neutrophil chemotaxis & cytokine production/release -Increases efferocytosis of apoptotic neutrophils by macrophages -Modulates inflammatory mediator release -Promotes fibroblast proliferation & skin wound healing -Shows no signs of toxicity or genotoxicity	Impairing neutrophil chemotaxis & cytokine production can reduce excessive inflammation, fostering a more anti-inflammatory environment that supports efferocytosis.-Enhancing the efferocytosis of apoptotic neutrophils by macrophages can promote the efficient clearance of these inflammatory cells.	Inflammation, skin wound healing	In vitro (neutrophils, macrophages), in vivo (animal model)
Pomegranate Peel extract [212]	*Allium cepa* (Onion), *Malus domestica* (Apple), Vitis vinifera (Grape), *Vaccinium spp*. (Berries)	-Decreases plaque necrosis & increases lesional collagen content. -Improves plaque stability. -Favorable changes in metabolic parameters, (*e.g*., lower blood glucose, cholesterol, & triglyceride levels) -Enhances efferocytosis efficiency, through the efferocytosis receptor Mertk & blocking the shedding of Mertk.	By improving plaque stability, modulating metabolic parameters, enhancing macrophage efferocytosis via Mertk upregulation, & preserving Mertk function by blocking its shedding, these factors collectively support efficient clearance of ACs.	Atherosclerosis; advanced atherosclerosis progression	-*Apoe*^*-/-*^ mice; In vitro
Quercetin [172, 173, 213]	*Allium cepa* (Onion), *Malus domestica* (Apple), Vitis vinifera (Grape), *Vaccinium spp*. (Berries)	-Inhibits monocyte migration -Decreases the expression of ICAM-1 & MCP-1 -Increases cholesterol efflux -Prevents cell infiltration in atherosclerotic plaques -Reduces the risk of stroke or brain destruction by mediating the LXR/RXR signaling pathway. -Promotes M2 polarization.	-Inhibiting monocyte migration, creating a more favorable environment for efferocytosis. -Downregulating ICAM-1 & MCP-1, which recruit inflammatory cells, can promote an anti-inflammatory state that supports efferocytosis. -The LXR/RXR signaling pathway is involved in the regulation of inflammatory processes & lipid metabolism. -Promoting M2 polarization enhances macrophage efferocytic capacity & inflammation resolution.	Atherosclerosis, inflammation; - Osteoarthritis, chronic synovitis	-THP-1 macrophages; - In vivo (animal model)
Rubus imperialis extract & niga-ichigoside F1 compound [214]	Leaves of the Rubus imperialis plant; Fruit of Rubus coreanus Leaves of *Rubus imperialis*, *Rubus coreanus* (Fruit)	-Promotes reduction in the inflammatory process induced by LPS or carrageenan. -Reinforces NO reduction in LPS-stimulated neutrophils. -Increases efferocytosis. -Shows wound healing properties. -Exhibits scavenging activity for DPPH -Provides cytoprotection in H2O2-induced oxidative stress. -Niga-ichigoside F1 reduces NO secretion.	-Reducing NO production in LPS-stimulated neutrophils can help dampen the inflammatory response & support the resolution of inflammation & efferocytosis. - DPPH scavenging indicates antioxidant properties that help mitigate oxidative stress, creating a favorable environment for efferocytosis. - H2O2 induces oxidative stress, which impairs cellular function (*e.g*., efferocytosis).	Wound healing, inflammation	In vivo (mice), in vitro (L929 cells, neutrophils)
SFN [215]	*Brassica oleracea* (Broccoli), *Brassica rapa* (Chinese cabbage), *Brassica napus* (Kale)	-Decreased mycobacterial burden. -Activates efferocytosis. -Activation of efferocytosis was found to be caspase 3/7 independent but dependent on p38 MAPK signaling. -The induction of p38 MAPK is linked to the Nrf2 signaling pathway.	The activation of the p38 MAPK pathway, which regulates efferocytosis, is also linked to the Nrf2 signaling pathway. The connection between p38 MAPK & Nrf2 signaling indicates that modulating this pathway affects the efficiency of efferocytosis.	*Mycobacterium abscessus* (Mabs)	Human THP-1-derived macrophages
PCA [216]	*Olea europaea* (olives), *Hibiscus sabdariffa* (roselle), *Eucommia ulmoides* (du-zhong), *Citrus microcarpa Bunge* (calamondin), & *Vitis vinifera* (white wine grapes)	-Increases the continual efferocytic capacity of macrophages -Inhibits the progression of advanced atherosclerosis -Reduces intracellular amounts of miR-10b -Promotes miR-10b secretion in extracellular vesicles -Increases abundance of the miR-10b target KLF4 -Transcriptionally induces the gene encoding MerTK -Increases continual efferocytic capacity	-Reducing the intracellular levels of miR-10b enhances efferocytosis through the modulation of downstream target genes (*e.g*., KLF4). -MerTK is a key receptor involved in the recognition & engulfment of ACs during efferocytosis. The transcriptional induction of the MerTK gene can increase the expression of this efferocytosis receptor.	Advanced atherosclerosis	Mice, naive macrophages
Ginsenoside Rg5 [217]	*Panax ginseng*, *Panax quinquefolius* (Ginseng)	- Promotes wound healing. - Reduces the negative regulation of SLC7A11 on the efferocytosis of DCs. - Physically interacts with SLC7A11 & suppresses its activity. - Reduces NF-κB p65 & SLC7A11 expression in the wounded areas. - Reduces glycose storage & enhances anaerobic glycolysis in DCs.	-By reducing the negative regulation of SLC7A11 on DCefferocytosis, these factors can promote the efficient clearance of ACs by DCs. - The reduction in NF-κB p65 & SLC7A11 expression in the wounded areas suggests a potential anti-inflammatory & pro-efferocytosis effect.	Diabetic wounds	Mice, BMDCs, cDC1s
Celosins [120]	The active constituents extracted from *Celosia argentea* (Cockscomb).	- Reduced the prevalence of plaque in the aorta. - Promoted autophagy. - Reduced phagocytosis of macrophages & the formation rate of foam cells. -Down-regulates the expression of CD36 & SR-A1 genes. -Up-regulates the expression of ABCA1 & ABCG1 genes. -Increased the levels of autophagy-specific proteins LC3 & beclin 1.	- Excessive lipid phagocytosis by macrophages can lead to foam cell formation, impairing efferocytosis. -CD36 & SR-A1 are receptors that mediate the uptake of lipids, including Ox-LDL. -ABCA1 & ABCG1 are transporters involved in the efflux of cholesterol from cells.	Atherosclerosis	*ApoE*^*-/-*^mice, Foam cell model using peritoneal macrophages
Low concentration resveratrol [76]	*Vitis vinifera* (Grape), *Arachis hypogaea* (Peanut), *Vaccinium spp*. (Berries)	-Sirt1 & autophagy marker proteins were increased & decreased in the low & high nicotinamide groups. -Efferocytosis was highest in the resveratrol group & relatively lower in the low & high concentration nicotinamide groups. -Enhancing Sirt1-mediated autophagy improves the efferocytosis.	-Increase the levels of Sirt1 & autophagy-related proteins (such as LC3 & beclin-1), resulting in enhanced efferocytosis of ACs.	Atherosclerosis	RAW264.7 cells
Tnnin with honey [218]	*Citrus limon* fruit juice	-Shows antilipidemic & antioxidant activity. -Inhibits LDL oxidation, preventing foam cell development. -Inhibits proliferation & induced apoptosis.	Increasing OxLDL reduces eferocytosis by the formation of macrophage foam cells.	Atherosclerosis	RAW 264.7 & THP-1 cells
An extract of Scoparia dulcis [219]	*Scoparia dulcis*	-Shows potent antioxidant activity & scavenged H2O2. -Improves erythrocyte membrane stabilization. -Inhibits lipid peroxidation & LDL oxidation, preventing foam cell formation.	- Oxidative stress & the accumulation of ROS, such as H2O2, can impair cellular function & efferocytosis. - Erythrocytes can release signals that promote the clearance of ACs through efferocytosis.	Atherosclerosis	RAW 264.7 cells
Millet shell polyphenols [220]	Millet shell	-Inhibits lipid phagocytosis, reducing the formation of macrophage-derived foam cells. -Reduces the secretion of IL-1β & TNF-α by inhibiting STAT3 & NF-κB expression. -Promotes the transformation of HASMCs from synthesis to contraction, reducing the formation of SMC-derived foam cells. -Regulates the gene expression levels of SMMHC, desmin, smoothelin, & elastin. -Increased HDL-C.	-The inhibition of STAT3 & NF-κB in macrophages is a key mechanism, which can enhance efferocytosis. By reducing the secretion of IL-1β & TNF-α, a more favorable environment is established for the effective clearance of ACs by macrophages.	Atherosclerosis	Macrophages & HASMCs (cell lines), *ApoE*^*-/-*^ mice
Tanshinone IIA [221]	*Salvia miltiorrhiza Bunge* (Danshen)	-Reduces macrophage content, cholesterol accumulation, & atherosclerotic plaque development. -Inhibits foam cell formation induced by ox-LDL by reducing ox-LDL uptake & promoting cholesterol efflux. -Reduces the expression of SR-A & increases the expression of ABCA1 & ABCG1. -Activates of the ERK/ Nrf2/ HO-1 pathway.	- SR-A facilitates the uptake of modified lipoproteins, leading to foam cell formation. - ABCA1 & ABCG1 are cholesterol transporters that promote cholesterol efflux from cells, preventing excessive lipid accumulation. -The ERK signaling pathway regulates transcription factors like Nrf2, which is a master regulator of antioxidant & cytoprotective genes, including HO-1. -The activation of the ERK/Nrf2/HO-1 pathway likely underlies how these factors modulate genes involved in lipid metabolism and efferocytosis.	Atherosclerosis	Human macrophages, *ApoE*^*-/-*^ mice
Curcumin [113–115, 136–139]	The rhizomes of the turmeric plant (*Curcuma longa*)	-Exhibits anti-inflammatory effects. -Has inhibitory effects on the activation of the NLRP3 inflammasome in macrophages. -Increases M2 phenotype & reduces lipid accumulation induced by Ox-LDL. -Modulates the microglial transcriptome, activates the Akt/Nrf2 pathway, & exhibits neuroprotective effects through Sirt1 signaling.	- The inhibition of the NLRP3 inflammasome, increasing anti-inflammatory M2 phenotype, the reduction in lipid accumulation, & the modulation of microglial function, contributing to creating a more favorable cellular environment for efficient efferocytosis.	Inflammatory conditions	*C57BL/6* mice, MCAO rats, rat cerebral cortical neurons, THP-1 monocytes/macrophages

*MerTK* Mer proto-oncogene tyrosine kinase, *KLF4* Krüppel-like factor 4, *LPS* Lipopolysaccharide, *PAI-2* Plasminogen activator inhibitor 2, *ALI* acute lung injury, *IBD* Inflammatory bowel disease, *H2O2* Hydrogen peroxide, *ROS* Reactive oxygen species, *Ox-LDL* Oxidized low-density lipoprotein, *LPC* Lysophosphatidylcholine, *Nrf2* Nuclear factor E2-related factor 2, *HO-1* Heme oxygenase-1, *CO* Carbon monoxide,*Mabs* Macrophages infected with Mycobacterium abscessus, *NK cells* natural killer cells, *MPMs* Murine peritoneal macrophages, *EGCG* Epigallocatechin gallate, *SFN* Sulforaphane, *PCA* Protocatechuic acid, *BMDCs* Bone marrow-derived dendritic cells, *cDC1s* Conventional type 1 dendritic cells, *TIPE2* Tumor necrosis factor alpha-induced protein 8-like 2, *TRPV1* Transient receptor potential vanilloid 1, *ICAM-1* Intercellular adhesion molecule-1, *Sirt1* Sirtuin 1, *MCP-1* Monocyte chemoattractant protein-1, *DPPH* 2,2-Diphenyl-1-picrylhydrazyl, *ACs* Apoptotic cells, *IBD* Inflammatory bowel disease, *FAF1* Fas-associated factor 1, *ALI* Acute lung injury, *Keap1* Kelch-like ECH-associated protein 1, *HDL-C* High-density lipoprotein cholesterol, *SMMHC* Smooth muscle myosin heavy chain, *ERK* Extracellular signal-regulated kinase, *NO* Nitric oxide, *Nrf2* Nuclear factor-erythroid 2-related factor 2, *HO-1* Heme oxygenase-1, *ABCA1* ATP-binding cassette transporter A1, *SR-A* Scavenger receptor, *HASMCs* Human aortic smooth muscle cells.

Enhancing autophagy may significantly improve AC recognition and clearance [66, 78]. Inhibiting autophagy by silencing autophagy-related gene 5 (ATG5) or other autophagy-related proteins increases apoptosis and oxidative stress mediated by NADPH oxidase. ATG5 is involved in the formation of autophagosome, cellular structures that encapsulate and degrade damaged or unnecessary cellular components. Additionally, this inhibition reduces the identification of ACs in efferocytes in macrophages lacking ATG5 [78]. In the context of microglia, the primary phagocytes in the brain, autophagy facilitates the efficient removal of dead cells, thereby preventing necrosis and subsequent inflammatory damage to healthy tissue [77].

When autophagy is disrupted, either in laboratory settings or in living organisms, there is a concomitant increase in apoptosis and a decreased ability of efferocytic phagocytes to recognize and engulf deceased cells [67, 76, 78]. This indicates that autophagy can trigger two distinct protective responses: promoting cell survival under normal conditions and facilitating efficient removal of cells by neighboring phagocytes when repair is not possible.

During the onset of atherosclerosis, when macrophages consume oxidized LDL (Ox-LDL) or other modified lipoproteins, they transform into foam cells. The buildup of these foam cells leads to the development of atherosclerotic plaques. Autophagy in macrophages helps prevent foam cell formation by decreasing the ingestion of Ox-LDL and enhancing both efferocytosis and cholesterol efflux. Efficient cholesterol efflux is important for maintaining macrophage function and their ability to effectively perform efferocytosis. [79, 80]. Moreover, defects in autophagy can lead to impaired efferocytosis, resulting in the accumulation of ACs and increased inflammation, as seen in conditions, such as stroke and neurodegeneration [74, 75, 77]. In therapeutic contexts, strategies aimed at boosting autophagy may enhance the ability of immune cells, like macrophages, to clear ACs effectively. This approach could be particularly beneficial in diseases characterized by defective efferocytosis, such as atherosclerosis and IBD, where improved clearance of dead cells can mitigate inflammation and promote tissue repair [66, 78]. Therefore, the interplay between autophagy and efferocytosis has emerged as an important area of research for understanding the regulation of inflammation and tissue homeostasis.

## Phytochemicals: nature’s toolbox for efferocytosis enhancement

### Phytochemical diversity and sources

Phytochemicals, naturally occurring compounds found in plants, have shown great potential for enhancing efferocytosis, the process of clearing ACs. These diverse plant-derived molecules possess various bioactive properties, including antioxidant, anti-inflammatory, and antimicrobial activities [29, 30]. Phytochemical treatments may offer advantages over conventional medications, such as a more natural and holistic approach, broad health benefits, potential synergistic effects, and fewer side effects [29, 30]. The mechanisms by which various phytochemicals modulate the key factors and pathways related to efferocytosis in various disease conditions are summarized in the Table 2. This table provides a comprehensive overview of the potential of phytochemicals to enhance efferocytosis and its therapeutic implications. By consolidating the existing knowledge on phytochemical-mediated modulation of efferocytosis, this chapter aims to offer valuable insights for future research and clinical applications, particularly in the context of chronic inflammatory and autoimmune disorders.

Conventional treatments for efferocytosis-related disorders often have limited efficacy and are associated with adverse side effects. For example, in the context of atherosclerosis, standard therapies, such as statins and anti-inflammatory drugs have had a modest impact on disease progression due to their inability to fully restore defective efferocytosis [81]. In this line, phytochemicals (alone or in combination with other treatments) have demonstrated the ability to modulate key pathways and cellular processes involved in efferocytosis, such as regulating phagocytic activity, macrophage polarization, and autophagy. This multifaceted approach to enhancing efferocytosis and resolving inflammation is an area of growing interest [30, 82–90].

### Mechanisms of phytochemical-mediated enhancement of efferocytosis and autophagy

The different mechanisms through which various phytochemicals mediate the factors and pathways related to efferocytosis in different conditions/disorders are summarized in Table 2. As shown in Table, there is great potential for enhancing the efferocytosis properties of phytochemicals in disorders.

## Efferocytosis and autophagy dysregulation under pathophysiological conditions and its implications

### Modulation of efferocytosis and autophagy by phytochemicals in atherosclerosis

Atherosclerosis, the main cause of cardiovascular and cerebrovascular illnesses, is also a major contributor to other ailments, such as cerebral infarction and chronic cerebral insufficiency. It is commonly regarded as a disease characterized by cholesterol accumulation and inflammation caused by lipids [4]. This accumulation is believed to release pro-inflammatory cytokines and formation of cholesterol microcrystals within cells, activating the inflammasome [91]. Macrophages filled with cholesterol, known as “foam cells,” are prone to death, releasing their contents and potentially impairing efferocytosis, which can worsen inflammation [92] (Fig. 2).

Efferocytosis, the clearance of ACs, is crucial for resolving inflammation in atherosclerosis. This process helps prevent the buildup of ACs and the release of inflammatory substances [93–95]. Failing efferocytosis can lead to necrosis and the release of pro-inflammatory factors, creating a detrimental cycle that exacerbates atherosclerosis. Factors like TNF-α play a significant role in this process by promoting inflammation and inhibiting pathways that facilitate AC clearance [96, 97].

The ingestion of extracellular components including lipids, carbohydrates, proteins, and nucleic acids occurs as a result of AC engulfment. To manage the increased metabolic burden, macrophages must engage in degradation and efflux pathways, which are critical for reducing inflammation and promoting tissue repair [98–100]. Experiments in animals lacking TIM-4, MertK, milk fat globule-EGF factor 8 protein (MFGE8), or protein S (ProS) revealed reduced AC clearance, increased inflammation, and worsened atherosclerosis. As defective efferocytosis accelerates macrophage apoptosis, the necrotic core enriched with lipids expands as atherosclerotic plaque progression occurs [101–104].

Several variables affect vulnerable plaques and acute coronary artery syndrome, including decreased fibrous cap formation, the presence of high inflammatory cytokine levels, intimal cell death, and the growth of the lipid-laden necrotic core. Furthermore, the lack of efferocytosis signals hinders the following pathways involved in the reverse transfer of intracellular cholesterol, allowing foam cells to form and the initiation of atherosclerosis to begin (Fig. 2). Notably, C1q protects against atherosclerosis by enhancing macrophage survival and improving the activity of foam cells in the early stages [105–107]. The interplay between autophagy and efferocytosis is particularly important in macrophages within atherosclerotic plaques. Autophagy enhances efferocytosis efficiency by providing the necessary resources for phagocytes to effectively engulf and process ACs. For instance, autophagy-related proteins can promote the formation of phagophores that facilitate the engulfment of apoptotic debris. Additionally, autophagy can regulate the expression of surface receptors involved in efferocytosis, thereby improving AC recognition and clearance. Conversely, impaired autophagy can lead to defective efferocytosis, resulting in the accumulation of ACs and a heightened inflammatory response. This dysregulation contributes to the progression of atherosclerosis and increases the risk of cardiovascular events [65, 76, 78, 79].

Experimental findings have provided evidence for the regulatory function of extracellular signal-regulated kinase 5 (ERK5) during macrophage efferocytosis. ERK5, a member of the mitogen-activated protein kinase (MAPK) family, is essential for maintaining macrophage phagocytosis and slowing the development of atherosclerosis [94]. In *LDLR*^*−/−*^ mice (a genetically modified mouse model that is deficient in the LDL receptor gene and is commonly used in atherosclerosis research), the elimination of the *ERK5* gene intensifies atherosclerosis and inhibits the expression of proteins associated with efferocytosis. Moreover, the use of an ERK5 inhibitor reduces the phagocytic activity of RAW264.7 cells (a macrophage cell line) in laboratory settings. Consequently, it can be inferred that the regulation of macrophage efferocytosis via ERK5 can be beneficial for combating atherosclerosis [108]. According to the data, there is a rising emphasis on treatment techniques that directly target the atherosclerotic macrophage phenotype in order to enhance disease regression. Various compounds originating from natural sources may provide a great possibility in this area (Table 2). These molecules possess multiple functions, including antioxidant properties, the ability to lower lipid levels, and the capacity to modulate cell signaling. As a result, they are likely to effectively counteract the development of lesions and, more importantly, in regulating the inflammatory response of macrophages.

Anti-atherogenic compounds found in nature, such as phytochemicals, have the ability to modify the pharmacological targets of trained immunity. Epigallocatechin gallate (EGCG) has been shown in studies to have antibacterial, antiviral, antioxidant, anti-vascular proliferation, anti-arteriosclerosis, anti-thrombosis, anti-inflammatory, and anti-tumor properties [109] (Table 2). EGCG also demonstrated inhibitory effects on the overexpression of type A scavenger receptor (SR-A) induced by Ox-LDL in the identical cell line, resulting in a reduction in the absorption of Ox-LDL and the formation of foam cells [110]. Curcumin has been demonstrated to regulate inflammation in both in vitro and in vivo studies. This characteristic renders curcumin a highly efficient remedy for inflammatory conditions, such as rheumatoid arthritis [111, 112]. The inhibitory effect of curcumin on the activation of NLR family pyrin domain containing 3 (NLRP3) inflammasome in macrophages has been established through numerous scientific investigations. This inhibitory effect is critical for preventing HFD-induced insulin resistance and the activation of the LPS-priming and NLRP3 inflammasome pathways in macrophages [113]. When it comes to controlling M1/M2 macrophages, curcumin can increase the release of M2 markers (*e.g*., macrophage mannose receptor (MMR), arginase 1, PPAR-, IL-4, and/or IL-13). Curcumin causes a shift in M1 macrophages towards an M2 phenotype in autoimmune myocarditis (EAM) and hyaline membrane disease in vivo [114]. A prior investigation revealed that curcumin significantly decreased the accumulation of lipids induced by Ox-LDL in J774.A1 macrophages. This reduction occurred by diminishing Ox-LDL uptake through SR-A and by enhancing cholesterol efflux *via* ATP binding cassette transporter A1 (ABCA1). Notably, curcumin did significantly affect other cholesterol transporters [115]. Among its multiple mechanisms, resveratrol plays a crucial role in protecting against atherosclerosis by regulating the differentiation of monocytes and macrophages. Furthermore, it inhibits the oxidation of LDL, enhances the protection of endothelial cells, reduces the levels of trimethylamine N-oxide (TMAO) through the modulation of gut flora, and hinders the proliferation and migration of vascular smooth muscle cells (VSMCs) [116]. Resveratrol effectively inhibited the formation of “foam cells” in RAW264.7 cells stimulated by LPS. This inhibition was achieved by reducing ROS production and MCP1 expression through the activation of the Akt/Forkhead Box O3a (Foxo3a) and AMPK/sirtuin 1 (Sirt1) pathways. Notably, the involvement of NADPH oxidase 1 (Nox1) was crucial for the aforementioned pathways to exert their effects [117, 118]. Furthermore, quercetin, a naturally occurring compound, has the ability to mitigate the development of atherosclerosis by modulating the occurrence of endothelial cellular senescence induced by Ox-LDL [119].

The potential of phytochemical-rich compounds in treating atherosclerosis by focusing on their ability to induce effective autophagy (not excessive) and efferocytosis. Specifically, the neuroprotective effects of berberine, 6-gingerol, and celosins through autophagy induction are evident in important disorders [74, 75, 120, 121]. Berberine activates autophagy through AMPK signaling, reducing inflammation induced by Ox-LDL, and improving the bacterial killing ability of macrophages [122, 123]. 6-gingerol exhibits neuroprotective effects by inhibiting inflammation, apoptosis, and oxidative stress through autophagy activation. It protects against brain ischemia/reperfusion damage and apoptosis caused by 6-hydroxydopamine [74]. Celosin inhibits atherosclerosis and promotes autophagy flow. In atherosclerosis models, celosins reduced the plaque prevalence and increased the number of autophagy bodies. In-vitro experiments demonstrated reduced phagocytosis and foam cell formation, along with increased of autophagy-specific protein expression [120].

Additionally, Sirt1 can enhance the clearance of Ox-LDL-induced ACs by macrophages through autophagy activation. Stimulating Sirt1 with a low concentration of resveratrol increased the expression of Sirt1 and autophagy markers, leading to improved efferocytosis. Conversely, inhibiting Sirt1 with nicotinamide decreased efferocytosis [76]. Further research in this area could provide valuable insights into developing more effective treatments for atherosclerosis by improving efferocytosis through autophagy modulation.

Furthermore, curcumin, ginger, and magnolol were the three natural medications studied. To modulating the medication release, a biodegradable polymer with varying molecular weights was utilized as a carrier for the medicines. In-vitro experiments revealed that all three medicines exhibited a burst release followed by continuous release lasting up to 38 days. Ginger exhibited the best compatibility compared with magnolol and curcumin, which had less ideal compatibility and fell into the mild-to-severe blood toxicity category. Thus, ginger has potential as a drug-coating material for drug-eluting stents, but more in-vitro testing is needed to validate its suitability [124].

### The modulation of efferocytosis and autophagy by phytochemicals in brain disorders

Proper elimination of ACs is critical for maintaining homeostasis of the central nervous system (CNS) and allowing its recovery after damage. Efferocytosis is critical for avoiding inflammation and other pathological problems in cases of physiological AC mortality. When ACs are not cleaned properly, the integrity of the AC membrane is compromised, leading to the release of intracellular contents and eventual secondary necrosis. Microglia, which are principally responsible for efferocytosis, are critical for the removal of ACs from the CNS [40, 125, 126]. A variety of cell types, including neural crest cells, neural stem cells, and astrocytes, can function as efferocytes. However, these cells are often less effective than microglia. When the blood-brain or blood-spinal cord barrier is disrupted during trauma, monocytes originating from the blood penetrate the CNS. Under normal conditions, seeing astrocytes in the CNS is difficult since they are rapidly consumed by microglia due to the very effective process of efferocytosis [13, 127]. Nonetheless, because efferocytosis is controlled by a mix of age, inflammation, and certain genetic risk factors, it can contribute to the development of CNS disorders. Several studies have highlighted the importance of dysregulated microglial/macrophage activity in the etiology of numerous clinical disorders, such as Alzheimer’s disease (AD), stroke, amyotrophic lateral sclerosis (ALS), and Parkinson’s disease (PD). Poor efferocytosis results in inadequate AC elimination [128, 129]. As a result, the excessive buildup of ACs overwhelms the efferocytosis ability of microglia/macrophages, generating a negative feedback loop. Secondary necrosis of uncleared ACs aggravates this situation by causing the production of pro-inflammatory factors, intracellular molecules, and danger-related molecular patterns (DAMPs), which increase the level of damage [130, 131]. Hence, efferocytosis is significantly important for promoting neuronal survival and facilitating axonal regeneration. By inhibiting the pro-inflammatory release as well as antigenic autoimmune constituents, efferocytosis contributes to the clearance of apoptotic debris. The remarkable therapeutic potential of this process in the context of neurodegenerative diseases necessitates additional preclinical development to fully exploit its benefits [46].

Dietary phytochemicals can influence numerous aspects of neuroinflammation. The neuroprotective effects of these compounds have been observed to depend on both the dosage and duration of exposure, as indicated by several studies. The neuroprotective effects of phytochemicals are believed to be mediated through various mechanisms. While the antioxidant properties of dietary phytochemicals have been extensively studied, it has been demonstrated that these compounds cannot only scavenge free radicals in the brain but also specifically target stress-activated signaling pathways that play crucial roles in neuroprotection. Numerous studies conducted in laboratory settings as well as in living organisms have provided significant evidence indicating that a wide range of phytochemicals have the ability to influence the expression of various genes that encode proteins promoting cell survival. These proteins include antioxidant enzymes, neurotrophic factors, and anti-apoptotic factors [132–134].

Curcumin at a concentration of 20 μM has a significant effect on the microglial transcriptome. This effect results in the development of an anti-inflammatory and neuroprotective phenotype in LPS-activated microglia [135]. Curcumin, when present at concentrations ranging from 5 to 25 μM, exerts its neuroprotective effects through the activation of the Akt/nuclear factor erythroid 2-related factor 2 (Nrf2) pathway. Nrf2 is a transcription factor that plays a crucial role in the cellular response to oxidative stress and the regulation of genes involved in antioxidant and cytoprotective processes. Thus, Nrf2 plays a critical role in mediating the neuroprotective effects of curcumin against oxidative damage [136, 137]. The neuroprotective properties of curcumin are also attributed to its ability to modulate Sirt1. Recent findings suggest that Sirt1 signaling activation is linked to the neuroprotective effects of curcumin. Moreover, administering curcumin as a pre-treatment (at a dosage of 50 mg/kg) has been shown to reduce apoptosis, inflammation, and mitochondrial dysfunction in ischemic brain injury in vivo [138]. Curcumin, at concentrations of 5–10 μM, has demonstrated efficacy in stimulating FoxO3a activity within monocytes/macrophages. Hence, curcumin may have an anti-oxidative effect on the inflammatory cells of the vascular system [139].

6-gingerol derived from *Zingiber officinale* (ginger) has demonstrates significant effects on related disorders through modulation of autophagy and efferocytosis. In the context of cerebral ischemia/reperfusion injury, studies on adult male Sprague-Dawley rats have shown that the protective effects of 6-gingerol are mediated through its ability to enhance autophagy and facilitate the clearance of apoptotic neuronal cells [74, 75]. 6-gingerol inhibits the NLRP3 inflammasome and apoptosis by promoting the dissociation of transient receptor potential vanilloid 1 (TRPV1) from Fas-associated factor 1 (FAF1), which leads to the activation of autophagy. This process not only reduces cerebral infarct volume but also improves brain edema and neurological scores, effectively reversing brain histomorphological damage, 6-gingerol upregulates autophagy by reducing inflammation derived from the NLRP3 inflammasome and neuronal apoptosis, thereby enhancing cellular survival and function [74, 75].

### The modulation of efferocytosis by phytochemicals in respiratory disorders (e.g., COPD and cystic fibrosis)

In lung inflammatory diseases, the rate of programmed cell death (apoptosis) is elevated, or the removal of these dead cells is impaired, resulting in cell accumulation [140]. This pathological process is primarily caused by the ineffective clearance of ACs by airway macrophages, a process known as efferocytosis [9]. Millions of people worldwide suffer from the degenerative lung disease known as chronic obstructive pulmonary disease (COPD). Oxidative stress and inflammation resulting from exposure to cigarette smoke or other environmental dangers play a significant role in the development of COPD [141]. Increased apoptosis and impaired clearance of ACs through a process called efferocytosis, contribute to chronic inflammation and tissue damage in patients with COPD [142].

Cystic fibrosis (CF) is caused by a mutation in the CF transmembrane conductance regulator (*CFTR*) gene. This genetic defect is characterized by significant airway inflammation and the accumulation of apoptotic (dying) cells [143]. Efferocytosis, the process of phagocytosis (engulfment) of ACs, is a crucial regulator of inflammation. Efferocytosis prevents the progression of apoptosis to necrosis (cell death) and actively suppresses the release of various pro-inflammatory mediators, such as IL-8 [144]. *CFTR* deficiency disrupts efferocytosis in airway epithelial cells, primarily due to increased expression of RhoA, a negative regulator, rather than altered binding or receptor expression [143]. Inhibiting RhoA or its downstream Rho kinase can restore efferocytosis in these cells. Additionally, an amiloride-sensitive ion channel is involved, as amiloride can improve phagocytic function in *CFTR*-deficient cells. This impaired efferocytosis leads to pro-inflammatory effects, with ACs boosting IL-8 release in CFTR-deficient cells but not in those with normal CFTR [145].

### The modulation of efferocytosis by phytochemicals in autoimmune-related disorders

Defective efferocytosis has emerged as a common pathogenic mechanism contributing to the chronic inflammation and tissue damage observed in autoimmune disorders, such as MS, T2D, and Crohn’s disease [146]. In an autoimmune encephalomyelitis (EAE) model of MS, inhibition of 12/15-lipoxygenase (12/5-LOX) by baicalein increased expression of PPARβ/δ in microglia. This was associated with reduced microglia activation, suppressed phagocytosis, and decreased production of pro-inflammatory cytokines and chemokines in the CNS. The modulation of efferocytosis and inflammatory pathways in microglia by 12/15-lipoxygenase inhibition attenuated the clinical severity of EAE, suggesting therapeutic potential in MS [147]. Moreover, defective efferocytosis has been linked to key pathological processes in T2DM, including pancreatic islet β cell destruction, skeletal muscle dysfunction, adipose tissue inflammation, and liver metabolism abnormalities [148]. The failure to effectively clear ACs leads to their accumulation and secondary necrosis, releasing of pro-inflammatory factors that contribute to the glucose homeostasis imbalance. Impaired efferocytosis is considered an initial node in development and progression of T2DM and its complications and is therefore a potential therapeutic target [148]. In a chronic mouse model of intestinal inflammation (SAMP/Yit), mesenchymal stem cell (MSC) therapy promoted mucosal healing and immunomodulation. The long-term therapeutic effects of MSCs were partially mediated by macrophage efferocytosis of apoptotic MSCs, leading to anti-inflammatory reprogramming of macrophages. This highlights the importance of efferocytosis in regulating the inflammatory response and promoting tissue repair in the context of chronic intestinal inflammation, as seen in Crohn’s disease [147]. Thus, modulating efferocytosis pathways represents a promising therapeutic approach for these autoimmune-related disorders by several phytochemicals, such as curcumin, crocin, PCA, and baicalein [146, 147, 149–151] (for more details see Table 3).

**Table 3:** Clinical trials investigating the safety and efficacy of phytochemicals in regulating inflammation and efferocytosis.

NCT number	Phyto chemical	Condition/Disorder (Sample Size)	Dose	Duration	Location	Phase(Status)	Results
NCT06003270	Quercetin	COPD (*n* = 30)	500 mg/day 1000 mg/day	Six Ms	United States	II (Recruiting)	No Results Posted
NCT01708278 [152]	Quercetin	COPD (*n* = 9)	500, 1000 or 2000 mg/day	One W	United States	1 (Completed)	-Quercetin was safely tolerated by COPD patients at doses up to 2000 mg per day for 1 week. -No severe adverse events were found related to the quercetin supplementation. -Only mild adverse events (*e.g*., gastro-oesophageal reflux disease, were observed in the placebo & quercetin groups).
NCT04851821	Quercetin	COVID-19 (*n* = 80)	NA	Ten Ds	Tunisia	1 (Completed)	No Results Posted
NCT04377789	QCB	COVID-19 (*n* = 447)	1000 mg quercetin	2–30 Ds	Turkey	Not Applicable (Completed)	- A greater decrease in CRP & ferritin levels. - A higher increase in platelet & lymphocyte counts. - The QCB group had more advanced pulmonary findings [222].
NCT01348204	Quercetin	CF (*n* = 32)	NA	NA	United States	II (Completed)	No Results Posted
NCT00402623	Quercetin	Sarcoidosis (*n* = 18)	1000 mg	24 Hs	Netherlands	Not Applicable (Completed)	-Improving the total plasma antioxidant capacity. -Reducing markers of oxidative stress (plasma malondialdehyde levels) & inflammation (plasma ratios of TNF-α/IL-10 & IL-8/IL-10) [156].
NCT04603690	Quercetin, curcumin & vitamin D3	SARS-CoV-2 (*n* = 50)	168 mg curcumin, 260 mg quercetin & 360 IU of vitamin D3	14 Ds	Pakistan	Not Applicable (Completed)	COVID-19-associated acute symptoms were resolved more quickly in the CUR-QUE group. The CUR-QUE supplementation was well-tolerated, & no treatment-emergent effects or serious adverse events were reported [223].
NCT02255370	Curcumin	Crohn’s disease (*n* = 61)	3 g/d	6 Ms	France	3 (Completed)	Curcumin was not more effective than placebo in preventing Crohn’s disease recurrence [149].
NCT00528151	Curcumin	LHON (*n* = 70)	500 mg/day	3, 6, & 12 Ms	Thailand	3 (Completed)	No Results Posted
NCT03122613	Curcumin	UC (*n* = 29)	2 g/day	12 Ms	Hong Kong	Not Applicable (Terminated)	No Results Posted
NCT00815763	Ginsenoside-Rd	Ischemic Stroke (*n* = 390)	20 mg	14 Ds	China	3 (Completed)	No Results Posted
NCT04163757	Crocin	Type 2 diabetes (*n* = 50)	NA	12 Ws	Iran	Not Applicable (Completed)	-Showing improvements in: - Fasting glucose level, HbA1C, Plasma insulin level, Insulin resistance, & Insulin sensitivity. - The active form of AMPK did not change significantly within or between the groups after the intervention [150].
NCT05696665	Apigenin Crocin	Neurodegenerative & Parkinson Disease (*n* = 120)	500 mg, 30 mg	NA	Pakistan	Not Applicable (Recruiting)	No Results Posted
NCT03470376	Monacolin K, berberine, policosanol 1, astaxanthin, folic acid & CoQ10	Hypercholesterolemia Inflammation Atherosclerosis (*n* = 26)	3, 500, 10, 0.5, 0.2 & 2 mg	3 Ms	Perugia	4 (Completed)	- No significant effects were observed for HDL-cholesterol, triglycerides, or lipoprotein(a). - The nutraceutical combination treatment was well-tolerated, with no significant alterations in muscle, liver, or immunovirological parameters. -Improving the lipid profile, PCSK9 levels, subclinical inflammation, and arterial stiffness in stable HIV-infected patients on antiretroviral therapy [224].
NCT05915117	Pomegranate	Metabolic Syndrome (*n* = 60)	500/mg	8 Ws	Bosnia & Herzegovina	Not Applicable (Active, not recruiting)	No Results Posted
NCT01269723	SFN	Smoking (*n* = 51)	NA	21 Ds	United States	Not Applicable (Completed)	-Promise as a safe, low-cost strategy for reducing influenza risk among smokers [225, 226]
NCT01181830	PCA	Metabolic Syndrome Family & Diabetes (*n* = 14)	8.1 mmol	NA	Italy	4 (Completed)	-Short-term ingestion of broccoli sprout homogenates can reduce virus-induced inflammation & viral replication in the nasal passages of smokers. -Nutritional antioxidant interventions with BSH have promise as a safe, low-cost strategy for reducing influenza risk among smokers & other high-risk populations [151].
NCT02998918	Purcumin & resveratrol	Inflammation/ Atherosclerosis/ CVD (*n* = 21)	500 mg	1 W	United States	Not Applicable (Completed)	No Results Posted
NCT01492114	Resveratrol	Chronic Subclinic Inflammation/ Redox Status (*n* = 40)	500 mg	30 Ds	Italy	3 (Completed)	No Results Posted
NCT01637675	Tanshinone IIA	CVD/ Lung Diseases (*n* = 90)	80 mg	8 Ws	China	2&3 (Unknown status)	Tanshinones, especially tanshinone I, were identified as cap-dependent endonuclease inhibitors with broad-spectrum antiviral effects [227].
NCT05130671	CQC	Mild Symptoms of COVID-19 (*n* = 50)	168 mg curcumin; 260 mg quercetin; 9 µg of cholecalciferol	2 W	Pakistan	Not Applicable (Completed)	-The CQC adjuvant therapy was safe & well-tolerated, with no treatment-emergent effects or adverse events reported. -The co-supplementation of CQC may have a therapeutic role in the early stage of COVID-19, helping with viral clearance, symptom resolution, & modulation of the inflammatory response [157].

*CF* Cystic Fibrosis, *CVD* Cardiovascular disease, *BSH* Broccoli sprout homogenates, *COPD* Chronic obstructive pulmonary disease, *HbA1C* Hemoglobin A1C, *LHON* Leber’s Hereditary Optic Neuropathy, *CUR-QUE* A combined curcumin and quercetin, *CQC* Curcumin, quercetin, and cholecalciferol, *PCSK9* Proprotein convertase subtilisin/kexin type 9, *PCA* Protocatechuic acid, *SARS-CoV-2* Severe Acute Respiratory Syndrome Coronavirus 2, *SFN* Sulforaphane, *QCB* Quercetin, vitamin C, and bromelain, *UC* Ulcerative colitis, *CRP* C-reactive protein; (*M* Months, *W* Week, *D* Day, *Hs* Hours).

## Clinical trials targeting efferocytosis-related factors/pathways

### Clinical studies investigating phytochemicals in efferocytosis-related pathologies

In 2020, a randomized clinical trial was conducted to examine the safety of quercetin supplementation in patients with COPD. The researchers enrolled individuals with varying degrees of lung disease. The results confirmed that there were no severe adverse events associated with the administration of the study drug, as determined by blood tests. However, it is worth noting that one patient reported experiencing mild gastro-oesophageal reflux disease (GERD) in both the quercetin and placebo groups. Overall, the study showed significant changes in blood profile, lung function, and the COPD assessment questionnaire indicated that quercetin was well-tolerated up to a dose of 2000 mg/day (NCT01708278) [152] (Table 3).

Furthermore, another randomized and double-blind clinical trial was conducted to evaluate the effectiveness of quercetin in the treatment of severe acute respiratory syndrome coronavirus 2 (SARS-COV2) patients. Although the trial recognizes the need for further understanding of quercetin, its antioxidant, anti-inflammatory, and antihistamine characteristics have been observed in numerous in vitro and animal investigations (NCT04377789) (Table 3).

Defects in the gene that encodes the CFTR have been identified as the cause of CF, predominantly affecting the pulmonary and digestive systems and resulting in early mortality owing to gradual deterioration in pulmonary function [144, 145]. In vitro studies have shown that quercetin can activate CFTR. The Nasal Potential Difference (NPD) test, which measures voltage across the nasal membrane, is an important biomarker of CFTR function in vivo [153]. In vitro and in vivo studies have revealed that quercetin activates CFTR in addition to the effects of existing NPD reagents [154, 155]. Furthermore, it has been discovered to activate rescued mutant *CFTR* in vitro, namely the ∆*F508 CFTR* mutation, which is the most prevalent cause of CF. The preliminary data suggests that the administration of quercetin may improve defective activation of the *ΔF508 CFTR* mutation, which is a common CF-causing mutation in which the CFTR protein is present on the cell surface. Therefore, the use of quercetin in the NPD protocol is likely to enhance the detection of the *ΔF508 CFTR* present at the cell surface.

This potential improvement in the detection of surface-localized *ΔF508 CFTR* represents a possible means to identify new candidates that may benefit from systemic CFTR potentiator therapy. Potentiator therapies aim to enhance the activity of CFTR channels, which can be beneficial for individuals with CF who have CFTR mutations that result in protein localization on the cell surface(NCT01348204) (Table 3).

Sarcoidosis, an inflammatory disease, is believed to be caused by oxidative stress and inadequate antioxidant levels. Oxidative stress, defined as an imbalance between ROS generation and the body’s ability to protect against them, plays an important role in the development of sarcoidosis. In individuals with sarcoidosis, the levels of antioxidants, which are crucial for defending against ROS, are lower. Consequently, antioxidant therapy aimed at strengthening the diminished antioxidant defense against sarcoidosis could be beneficial. Additionally, since ROS can initiate and mediate inflammation, antioxidant therapy may also alleviate the heightened inflammation observed in sarcoidosis. A clinical trial was conducted to evaluate the effects of a double-blind intervention on two groups of non-smoking, untreated sarcoidosis patients, according to this information. Malondialdehyde levels in plasma were examined as a sign of oxidative damage, while tumor necrosis factor (TNF)/IL-10 and IL-8/IL-10 ratios in plasma were assessed as indicators of inflammation. Quercetin supplementation was shown to be beneficial in sarcoidosis because it is a flavonoid with anti-inflammatory and anti-oxidative effects, making it a viable antioxidant treatment (NCT00402623) [156] (Table 3).

Another group of scientists investigated the benefits of a combination of dietary supplements, specifically quercetin, curcumin, and vitamin D3, as an extra remedy for the initial mild indications of COVID-19 infection in patients who are not hospitalized. The results revealed that these dietary supplements possessed potent antioxidant, anti-inflammatory/immunomodulatory, and extensive antiviral properties (NCT05130671) [157] (Table 3).

A clinical trial was conducted to examine the effects of curcumin in Crohn’s disease. This goal of the study was to determine if the combination of curcumin and thiopurine could effectively prevent the recurrence of Crohn’s disease following surgery (NCT02255370). This study revealed that oral curcumin was not more effective than placebo in preventing he of Crohn’s disease recurrence after surgery [149] (Table 3).

The efficacy of curcumin, a biologically active phytochemical present in turmeric, has been evaluated for the maintenance of remission in patients with ulcerative colitis (UC) [158, 159]. A randomized, double-blind, multicenter trial was conducted to specifically assess the potential benefits of curcumin as a maintenance therapy for patients with quiescent UC [158]. The trial enrolled 89 patients with quiescent UC, with 45 receiving curcumin (2 g/day) in addition to their standard treatment of sulfasalazine or mesalamine, and 44 receiving placebo plus the standard medications [158]. The study found that significantly fewer patients relapsed in the curcumin group than in the placebo group at the end of the 6-month treatment period [158]. Additionally, the use of curcumin was associated with improvements in the clinical activity index (CAI) and endoscopic index (EI), indicating a suppression of the morbidity associated with UC [158]. These findings were supported by a systematic review [159]. The review confirmed the beneficial effects of curcumin, reporting that 4% of patients in the curcumin group experienced relapse after 6 months, compared with 18% in the placebo group [159]. At 12-month follow-up, the relapse rates were 22% and 32% in the curcumin and placebo groups, respectively [159]. Consistent with the original trial, the systematic review also found that curcumin significantly improved the CAI and EI at 6 months compared with placebo [159].

## Future perspectives: expanding the frontiers of modulating efferocytosis by phytochemicals

### Emerging technologies and approaches for targeted efferocytosis modulation

#### Synergistic interactions of phytochemical combinations

The potential to understand how phytochemical combination interact holds great promise for maximizing health benefits and preventing chronic diseases. Understanding these pathways is important for maximizing synergistic effects while avoiding antagonistic effects in daily diets and phytochemical-based therapies for chronic disorders [160]. In a specific study, the combination of curcumin and piperine exerted synergistic effects on pain-like behaviors without any potential side effects on the CNS in mouse models. These findings suggest that this combination could be a safe and effective strategy for managing pain [161]. Other studies have also highlighted the immunomodulatory effects of phytochemical combinations, such as the synergistic enhancement of macrophage-mediated immune responses through the combination of vegetable soup and beta-glucan [162]. Another study showed increased phagocytic activity of macrophages through the synergistic actions of *Caulerpa racemosa* and *Eleutherine americana* extracts [163]. These findings offer valuable insights into the development of functional foods and phytochemical-based treatments for chronic diseases.

### Nanotechnology-enabled delivery systems for phytochemicals in efferocytosis modulation

Using nano-sized phytochemicals and spray technology for sublingual absorption can potentially improve the uptake and bioavailability of phytochemicals [164–166]. Nano-sized particles can enhance the surface area and penetration of the compounds, allowing for more efficient absorption through the sublingual mucosa. Sublingual administration involves placing the spray under the tongue, where it is directly absorbed into the bloodstream, bypassing the digestive system. This can accelerate the onset of action and avoid potential degradation or loss of efficacy in the gastrointestinal tract. However, it is important to note that the effectiveness and safety of nano-sized phytochemical sprays can vary depending on various factors, such as the specific formulation, dosage, and individual response [164, 165].

The combination of phytochemicals and nanotechnology has shown enormous potential for the long-term development and manufacture of nanotechnology-enabled goods. In one study, an aqueous extract of Olax nana Wall. ex Benth. was used to create biogenic silver and gold nanoparticles with biocompatibility and potential biomedical applications, such as anticancer, antibacterial, antileishmanial, antinociceptive, enzyme inhibition, and anti-inflammatory properties [167]. Similarly, ginseng-derived exosome-like nanoparticles (GENs) have been demonstrated to be effective in the treatment of colitis *via* modulating the intestinal microbiota and immune surrounding environment [168]. Curcumin-silver nanoparticles (Cur-AgNPs) produced from the phytochemical curcumin are efficient against Gram-positive and Gram-negative bacteria, while being less harmful to human keratinocytes [169]. Finally, liquid crystalline nanoparticles (LCNs) encapsulating berberine, a phytochemical with potent anti-inflammatory and antioxidant properties, showed potent anti-inflammatory and antioxidant activities in vitro in LPS-induced mouse RAW264.7 macrophages [170]. *Elaeagnus angustifolia* leaf extracts were used as a non-toxic source of reducing and stabilizing chemicals to create zinc oxide nanoparticles (ZnONPs). The ZnONPs demonstrated a variety of biological uses and were proven to be biocompatible when tested with human erythrocytes and macrophages [171].

A new treatment method for osteoarthritis that involves repolarizing synovial macrophages from the M1 to M2 phenotype employs apoptotic chondrocyte membrane-coated, quercetin-loaded metalorganic framework nanoparticles. These nanoparticles mimic efferocytosis, are easily phagocytosed by synovial macrophages, promoting M2 polarization and inhibiting chondrocyte apoptosis [172, 173]. These studies demonstrate the potential of combining nanotechnology with phytochemicals for various biomedical applications.

## Conclusion

This comprehensive review explored the potential of phytochemicals in modulating efferocytosis, the critical process of clearing ACs, and its implications for the prevention and treatment of inflammatory and autoimmune disorders. This review highlighted the central role of efferocytosis in maintaining immune regulation and tissue homeostasis. Dysregulation of this process is closely linked to the pathogenesis of various chronic inflammatory conditions, such as atherosclerosis, neurodegenerative diseases, and autoimmune disorders. By consolidating the current understanding of the mechanisms by which phytochemicals enhance efferocytosis, this review offers valuable insights for future research and clinical applications. Exploring phytochemicals as enhancers of efferocytosis, inflammation resolution, and wound healing presents new strategies for immune regulation and tissue balance. Defective efferocytosis is linked to chronic inflammatory and autoimmune disorders, emphasizing the need for therapeutic interventions. Phytochemical antioxidants from dietary sources show promise for increasing efferocytosis and autophagy, and may be effective against related conditions. They possess diverse bioactive properties and can reduce ROS, promote efficient removal of ACs, reduce inflammation, and induce autophagy. Understanding the potential of phytochemicals in efferocytosis-related disorders provides valuable insights for future research and clinical applications. Particularly for chronic inflammatory diseases like atherosclerosis, phytochemical-based interventions may offer new treatment avenues. Moreover, phytochemicals have neuroprotective properties and can influence gene expression, promoting cell survival. However, it is important to note that the effectiveness of phytochemical treatments can vary, and further research, including clinical trials and preclinical studies, is needed to optimize their use in therapeutic interventions. Further studies are recommended to fully elucidate the precise mechanisms of action and optimize the use of phytochemicals in therapeutic interventions, paving the way for their effective integration into clinical practice.

In conclusion, this review underscores the significant potential of phytochemicals as enhancers of efferocytosis and autophagy and modulators of inflammation resolution, offering new avenues for the management of chronic inflammatory and autoimmune disorders. Continued research in this field is promising for the development of effective, natural therapies that improve tissue homeostasis and overall patient outcomes.
